# Consecutive Evaluation of Systematic Community-Based COVID-19 Antigen Rapid Diagnostic Testing in Three Different Populations in Jamaica during the COVID-19 Pandemic

**DOI:** 10.4269/ajtmh.23-0883

**Published:** 2024-12-10

**Authors:** David Walcott, Chika Ozongwu, Carl Bruce, Alison Nicholson, Camille-Ann Thoms Rodriguez, Jerome Patrick Walker, John Lindo, Melanie Dawkins, Samantha Johnson, Kristen Collins, Tresana Pearson, Vanessa Bailey-Higgins, Pallavi Dani, Shaquielle Dias, Anne Hoppe

**Affiliations:** ^1^ Novamed, Kingston, Jamaica;; ^2^ University of the West Indies, Kingston, Jamaica;; ^3^ University Hospital of the West Indies, Kingston, Jamaica;; ^4^ FIND, Geneva, Switzerland;; ^5^ Elizabeth Glaser Pediatric AIDS Foundation, Geneva, Switzerland

## Abstract

The rise in COVID-19 cases in late 2021 posed a grave threat to the public health system and the economy of Jamaica. A key pillar of controlling COVID-19 includes rapid diagnosis of SARS-CoV-2 infected individuals and their contacts. Hence, we evaluated the feasibility and acceptability of weekly deployment of antigen rapid diagnostic tests (Ag-RDTs) by conducting three 6-week studies within high-risk populations in Jamaica. We enrolled 1) 287 study participants (≥18 years) from low-income communities (Study A), 2) 262 healthcare workers (Study B), and 3) 88 students (14–17 years) (Study C). Conducting these independent studies was challenging. Willingness to participate was generally low with fear of phlebotomy (42%), discomfort associated with nasal swab (39%), and lack of parental consent (35%) being the most common reasons students gave (Study C) for lack of participation. Furthermore, only 57%, 66%, and 88% of participants concluded their final study visit in studies A to C, respectively. Participants’ commitment and external factors, such as severe weather and outbreaks of violence affected follow-up. Overall, a total of six participants (<1%) tested COVID-19 Ag-RDT positive during all three studies, thus the number of infections detected were too low to draw any conclusions relating to the efficacy of the testing approach. Antibodies against SARS-CoV-2 were detected in most study participants (78–94%), but vaccination rates differed significantly between communities. Understanding these differences in vaccination rates is important because, given the low participation and follow-up rate, mass vaccination may present a more suitable public health intervention than regular testing.

## INTRODUCTION

The COVID-19 pandemic put significant strain on Jamaica’s public health system and the economy. In 2020, Jamaican public hospitals exceeded their capacity, forcing them to limit their services to emergency care only to accommodate the COVID-19 related admissions.^1^ Additionally, the tourism sector, which significantly contributed to the economy by accounting for more than 30% of the country’s gross domestic product—totaling more than 13 billion USD in 2020—was severely impacted by the pandemic.^2^^–^^4^ Travel restrictions led to a 10% economic decline in 2020, compared with a small 1% drop in 2019.^2^ Therefore, controlling COVID-19 was essential for both the well-being of the Jamaican people and the recovery of the Jamaican economy.

Kingston, the capital of Jamaica, is located on the southeast of the island. In the early 1900s, it amalgamated with St. Andrew, and together they have a combined population of just under 700,000 people, many of whom are impoverished and reside in inner cities and ghettos.^5^^–^^7^ Trench Town, located in the parish of St. Andrew, and August Town, located in Kingston, are two such inner-city communities with populations of more than 10,000 persons. In Trench Town, the estimated monthly income of the average resident is less than $25,000 JMD (approximately $160 USD), and in August Town most residents are unemployed.^8^^–^^10^ There are several public schools for children and adolescents, and communities have access to public health facilities in both communities.^8^^,^^11^^,^^12^ Residents in Trench Town reportedly have good health-seeking practices; however, the interactions with healthcare facilities and workers are generally rated negatively due to provider attitudes and waiting times.^8^ Gang-related crime and violence is a major concern in both communities, which complicates the ability to reach them for research purposes.^8^^,^^9^

Rapid diagnosis of COVID-19 was the first step in breaking the chain of transmission during the pandemic because it allowed for swift isolation of positive cases and contact tracing and proved valuable to do in both asymptomatic and symptomatic persons.^13^^,^^14^ Antigen rapid diagnostic tests (Ag-RDTs) provide diagnostic information within 15 minutes, can be performed within community settings, are highly specific, and are inexpensive.^14^^,^^15^ One study showed COVID-SMART (Systematic Meaningful Asymptomatic Repeated Testing) a pilot intervention, conducted in the United Kingdom, where asymptomatic persons voluntarily underwent rapid antigen testing, reduced the number of COVID-19 hospital admissions by 43%.^16^ However, to conduct mass testing successfully, public trust, proper quarantining, and social and organizational factors that encourage participation and contact tracing must be prioritized.^14^ The effective deployment of these tests was especially important in countries like Jamaica where return of polymerase chain reaction (PCR) results would take several days and testing and vaccination rates were low.^17^

We evaluated the impact of systematic community-based COVID-19 antigen rapid diagnostic testing on community transmission using the same testing strategy across three high-risk populations. These include 1) healthcare workers, who face risks due to fluctuating supply of personal protective equipment (PPE) and frequent contact with infected patients; 2) adults in low-income community households, who often face overcrowded and substandard housing conditions coupled with low health literacy and are thus at a higher risk of contracting SARS-CoV-2; and 3) students, who may struggle to practice social distancing in school and/or the home.^18^^–^^21^ The studies took place between March 2022 and October 2022. During this time, the proportion of Jamaicans who were vaccinated was estimated to be less than 25%, and the percent positivity was variable throughout the study with some days in June and September going above 20%. However, during most of the testing period, it remained below 5%, with the number of cases also decreasing during the time.^17^^,^^22^^–^^24^

## MATERIALS AND METHODS

### Populations and design of the three independent studies.

**Figure 1.**
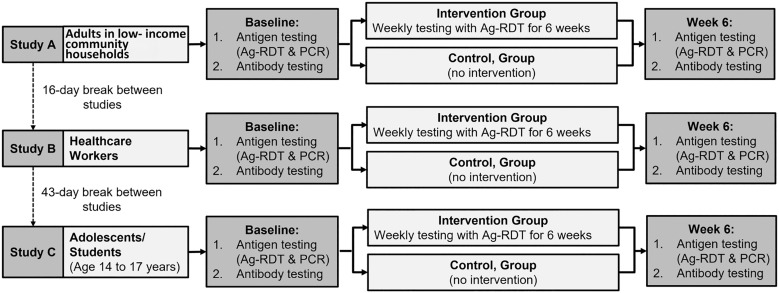
Study design.

We conducted three consecutive studies in different high-risk populations in Jamaica: 1) adults in low-income community households (Study A), 2) healthcare workers (Study B), and 3) adolescents attending secondary schools (Study C). Our goal was to evaluate the impact of systematic community-based testing with COVID-19 Ag-RDTs in these populations. Testing of the three high-risk populations was performed consecutively starting with adults in low-income community households, then healthcare workers, and finally adolescents (ages 14–17 years) (Figure 1).

Participants were recruited from communities in Kingston (Trench Town) and St. Andrew (August Town), Jamaica, for studies A and C. National data were used to identify communities as well as public and private schools within both townships to ensure that these were comparable in percent positivity, demographics, and socioeconomic profile. Study B was conducted at the University Hospital of the West Indies (UHWI), which is located in St. Andrew and borders August Town.

Each of the studies consisted of two study groups: an intervention group and a control group. Intervention and control groups were matched regarding key characteristics, including equal distribution of age, sex, comorbidities, socioeconomic factors, general risk of exposure, and vaccination status, then assigned to groups.

The communities of studies A and C were cluster randomized into intervention and control groups. Students were recruited from the same townships as participants in study A, therefore an approximately 4 month “wash-out” phase was applied between these studies to reduce the number of confounding factors impacting study C. For study B, healthcare workers of the UWHI, a tertiary institution, were randomly divided into test and control groups by stratified sampling, based on the demographics mentioned along with specialty. It was assumed that certain specialties had increased risk of COVID-19 exposure.^25^ The project was not designed to compare studies A, B, and C, but to individually assess the potential impact of each approach.

Testing with COVID-19 Ag-RDTs was performed weekly in the intervention groups and at baseline and week 6 in the control groups. Confirmatory PCR testing was performed at baseline and week 6. Antibody testing at baseline and at week 6 was performed to obtain data on exposure to SARS-CoV-2 or its vaccine at baseline and infection rates within the 6-week period.

The control groups served to evaluate the impact of the testing strategy on community transmission, should there be a surge in SARS-CoV-2 transmission at the time the different studies were conducted.

### Recruitment and testing procedure.

The communities in which testing was conducted were sensitized to the different studies via flyers and engaging with community leaders (Study A), hospital leadership (Study B), and staff at participating schools (Study C), along with word of mouth through the aforementioned interested participants and leaders from each group. Large-scale awareness campaigns were not conducted.

Individuals aged 18 and older (Studies A and B) and public secondary school students from ages 14 to 17 years (Study C) were invited to participate. Individuals were excluded from the study if they had a recent history of uncontrolled bleeding, were on anticoagulant therapy, had ear/nose/throat surgery or neurosurgery in the previous 12 months, had a serious underlying disease, or had a life expectancy less than 6 months.

Written informed consent was obtained from all study participants; for minors (<18 years), written assent was also obtained.

Compensation was given to participants in various forms. Specifically, participants in study A who had to travel received J$500 (∼3 USD) per trip to cover their cost for transportation, whereas those in studies B and C received refreshments (juice, fruits, and snacks) at the point of testing.

Sample collection and testing by COVID-19 Ag-RDTs (Standard Q COVID-19 Ag Test from SD Biosenso, Seongnam, South Korea) was done within mobile testing centers. At baseline and week 6, PCR testing was performed on all individuals using COVID-19 TaqMan RT-PCR Detection Kit (E/RdRP genes, Charite/Berlin protocol). Testing was carried out irrespective of symptoms. Antibody testing was carried out retrospectively at the UHWI using AdviseDx SARS-CoV-2 IgG II Quant assay to confirm recent infection of any individuals who had not been vaccinated. Antibody titers ≥50 AU/mL were reported as positive/detectable and titers <50 AU/mL were reported as negative/undetectable. All tests were done by trained staff from the UHWI as per the manufacturers’ instructions.

Study participants received an explanation of their results and information on infection prevention from a study team member qualified as a medical doctor or registered nurse. Reporting of positive cases and contact tracing was coordinated between the Ministry of Health and the UHWI. All individuals testing positive via either PCR or Ag-RDT (or both) were encouraged to self-isolate and referred to their nearest health center to facilitate reporting of the disease and further treatment as needed.

### Data collection and storage.

Data on exposure risk for SARS-CoV-2 infection (e.g., overseas travel, contact with confirmed case), flulike symptoms and others (e.g., diarrhea and vomiting), demographic data (age, sex) were collected for all three high-risk populations. All data was stored in a password-protected Microsoft Excel file on a designated computer and backed-up on an encrypted and securely stored hard drive.

### Student survey.

Adolescents age (ages 14–17 years) attending the schools where study C was conducted were invited to participate in a voluntary, fully anonymized opinion survey to obtain feedback on the recruitment approach and study experience (for those who participated in the study) and to obtain an insight into vaccine hesitancy (for all participating students) and reasons for the lack of interest in participating in this study (for those who did not participate in the study). Parents were informed about this voluntary survey. No personal data were collected, and responses cannot be linked to individual students. Missing information was handled as “information not disclosed.”

## STATISTICAL ANALYSES

The study was powered to detect statistically significant differences between intervention and control communities at a transmission rate of 5%. With transmission rates below 5%, we were not able to perform any analysis in this regard. Still, *t*-tests, binary logistic regressions, and χ^2^ tests were performed for key parameters to evaluate statistically significant differences between intervention and control groups and to assess which factors were most likely to influence follow-up behavior.

## RESULTS

### Demographics of study participants.

In total, 287 individuals (≥18 years) from low-income community households participated in study A (March 21, 2022 to April 3, 2022), 262 healthcare workers participated in study B (April 19, 2022 to July 7, 2022), and 88 adolescents (ages 14–17 years) enrolled into study C (August 19, 2022 to October 28, 2022).

At baseline, the control and intervention communities in study A were reasonably well matched with regard to basic factors such as vaccination status and symptoms as well as risk factors (Table 1). Similarly, healthcare workers in both the control and intervention groups were closely matched based on vaccination status, sex, and behaviors such as smoking status (Table 1) and their professional roles. In study C, however, although participants were closely matched based on sex and behaviors, a statistically significant difference was noted in the vaccination status of adolescents in the control group compared with the intervention group (*P* <0.001). Ninety-one percent (91%) of adolescents in the control group were vaccinated, whereas only 40% in the intervention group had been vaccinated against COVID-19 (Table 1).

**Table 1:** Demographics of control and intervention group at baseline (week 0) and week 6

Demographics	Week 0				Week 6			
	Total, *N* (%)	Control, *n* (%)	Intervention, *n* (%)	*P*-Value	Total, *N* (%)	Control, *n* (%)	Intervention, *n* (%)	*P*-Value
Study A: Within adults in low-income households								
Total	287 (100)	140 (100)	147 (100)	–	166 (57.8)	71 (50.7)	95 (64.6)	–
Sex								
Male	154 (53.7)	73 (52.1)	81 (55.1)	0.615	87 (52.4)	38 (53.5)	49 (51.6)	0.804
Female	133 (46.3)	67 (47.9)	66 (44.9)		79 (47.6)	33 (46.5)	46 (48.4)	
HCW in household								
Yes	35 (12.2)	20 (14.3)	15 (10.2)	0.506	13 (7.8)	9 (12.7)	4 (4.2)	0.064
No	249 (86.8)	119 (85.0)	130 (88.4)		152 (91.6)	61 (85.9)	91 (95.8)	
Unsure	3 (1.0)	1 (0.7)	2 (1.4)		1 (0.6)	1 (1.4)	–	
Vaccination status								
Vaccinated	123 (42.9)	59 (42.1)	64 (43.5)	0.811	76 (45.8)	33 (46.5)	43 (45.3)	0.876
Unvaccinated	164 (57.1)	81 (57.9)	83 (56.5)		90 (54.2)	38 (53.5)	52 (54.7)	
Smoker								
Yes	125 (43.6)	58 (42.3)	63 (44.1)	0.792	66 (39.8)	25 (35.2)	41 (43.2)	0.301
No	159 (55.4)	79 (57.7)	80 (55.9)		100 (60.2)	46 (64.8)	54 (56.8)	
Chronic disease								
Yes	81 (28.2)	40 (28.6)	41 (27.9)	0.560	52 (31.3)	21 (29.6)	31 (32.6)	0.247
No	202 (70.4)	97 (69.3)	105 (71.4)		112 (67.5)	48 (67.6)	64 (67.4)	
Unsure	4 (1.4)	3 (2.1)	1 (0.7)		2 (1.2)	2 (2.8)	–	
Public transportation								
Yes	253 (88.2)	122 (87.1)	131 (89.1)	0.382	153 (92.2)	64 (90.1)	89 (93.7)	0.401
No	30 (10.5)	17 (12.1)	13 (8.8)		13 (7.8)	7 (9.9)	6 (6.3)	
Elderly in household								
Yes	72 (25.1)	34 (24.5)	38 (26)	0.954	44 (26.5)	15 (21.1)	29 (30.5)	0.162
No	213 (74.2)	105 (75.5)	108 (74)		121 (72.9)	56 (78.9)	65 (68.4)	
Study B: Among HCWs								
Total	260 (100)	135 (100)	125 (100)	–	174 (66.9)	92 (68.1)	82 (65.6)	–
Sex								
Male	89 (34.2)	44 (32.6)	45 (36)	0.563	57 (32.8)	29 (31.5)	28 (34.1)	0.713
Female	171 (65.8)	91 (67.4)	80 (64)		117 (67.2)	63 (68.5)	54 (65.9)	
Vaccination status								
Vaccinated	218 (83.9)	113 (83.7)	105 (84)	0.948	154 (88.5)	81 (88)	73 (89)	0.840
Unvaccinated	42 (16.1)	22 (16.3)	20 (16)		20 (11.5)	11 (12)	9 (11)	
Smoking status								
Smoker	33 (12.7)	12 (8.9)	21 (16.8)	0.056	23 (13.2)	10 (10.9)	13 (15.9)	0.333
Nonsmoker	227 (87.3)	123 (91.1)	104 (83.2)		151 (86.8)	82 (89.1)	69 (84.1)	
Chronic disease								
Yes	58 (22.3)	33 (24.4)	25 (20)	0.390	51 (29.3)	30 (24.4)	21 (25.6)	0.311
No	202 (87.7)	102 (75.6)	100 (80)		123 (70.7)	62 (75.6)	61 (74.4)	
Public transportation								
Yes	43 (16.5)	25 (18.5)	18 (14.4)	0.372	23 (13.2)	15 (16.3)	8 (9.8)	0.203
No	217 (83.5)	110 (81.5)	107 (85.6)		151 (86.8)	77 (83.7)	74 (90.2)	
Proximity to positive COVID-19 case								
Yes	72 (27.7)	39 (28.9)	33 (26.4)	0.654	38 (21.8)	21 (22.8)	17 (20.7)	0.739
No	188 (72.3)	96 (71.1)	92 (73.6)		136 (78.2)	71 (77.2)	65 (79.3)	
Elderly in household								
Yes	50 (19.2)	31 (23)	19 (15.2)	0.113	33 (19)	20 (21.7)	13 (15.9)	0.323
No	210 (80.8)	104 (77)	106 (84.8)		141 (81)	72 (78.3)	69 (84.1)	
Study C: Among adolescents (ages 14–17 years)								
Total	88 (100)	43 (100)	45 (100)	–	73 (83)	40 (93)	32 (71.1)	–
Sex								
Male	50 (56.8)	22 (51.2)	28 (62.2)	0.295	45 (61.6)	22 (55)	22 (68.75)	0.234
Female	38 (43.2)	21 (48.8)	17 (37.8)		28 (38.4)	18 (45)	10 (31.25)	
Vaccination status								
Vaccinated	57 (64.8)	39 (90.7)	18 (40)	<0.001	50 (68.5)	37 (92.5)	12 (37.5)	<0.001
Unvaccinated	31 (35.2)	4 (9.3)	27 (60)		23 (31.5)	3 (7.5)	20 (62.5)	
Smoking status								
Smoker	10 (11.4)	2 (4.7)	8 (17.8)	<0.052	11 (15.1)	4 (10)	7 (21.9)	0.164
Nonsmoker	78 (88.6)	41 (95.3)	37 (82.2)		62 (84.9)	36 (90)	25 (78.1)	
Chronic disease								
Yes	10 (11.4)	3 (7)	7 (15.6)	0.205	7 (9.6)	3 (7.5)	4 (12.5)	0.477
No	78 (88.6)	40 (93)	38 (84.4)		66 (90.4)	37 (92.5)	28 (87.5)	
Public transportation								
Yes	64 (72.7)	32 (74.4)	32 (71.1)	0.728	54 (74)	29 (72.5)	25 (78.1)	0.584
No	24 (27.3)	11 (25.6)	13 (28.9)		19 (26)	11 (27.5)	7 (21.9)	
Unsure	84 (95.5)	40 (93)	44 (97.8)		72 (98.6)	39 (97.5)	32 (100)	
Elderly in household								
Yes	13 (14.8)	3 (7)	10 (22.2)	0.044	8 (11)	3 (7.5)	5 (15.6)	0.276
No	75 (85.0.2)	40 (93)	35 (77.8)		65 (89)	37 (92.5)	27 (84.4)	

HCW = healthcare worker.

At week 6, participants were very closely matched in both the control and intervention groups for all categories except lifestyle (Study A) and vaccination status (Study C) (Table 1). In study A, males were significantly more likely to be smokers than females (*P* <0.001), and nonsmokers were more likely to be vaccinated (*P* = 0.044). Persons with a healthcare worker living in their homes were also more likely to be vaccinated (*P* = 0.004).

### Willingness to participate and reasons for low or nonparticipation in these studies.

Willingness to participate in any of the three studies was generally low, and significant sensitization of the different communities was required to reach a reasonable sample size. High levels of apprehension about being tested for COVID-19 and COVID-19 fatigue, testing being perceived as enforcing government policies, and the study being incorrectly associated with vaccination campaigns were the most common reasons expressed by individuals who did not want to participate in studies A and B.

Vaccination rates within the study populations were low among all groups except the healthcare workers (84% at baseline and 89% at week 6; Table 1) among whom vaccination was recommended and prioritized, but not compulsory.^26^ Contrastingly, less than 46% of the adults in low-income community households (Study A), and less than 68% of students (Study C) were vaccinated at the time the studies were conducted (Table 1). According to a vaccine uptake survey conducted in Jamaica in 2021, an estimated 30% of the population were hesitant about getting vaccinated against COVID-19, with safety and efficacy concerns presenting a key concern.^17^

### Follow-up during the studies.

**Figure 2.**
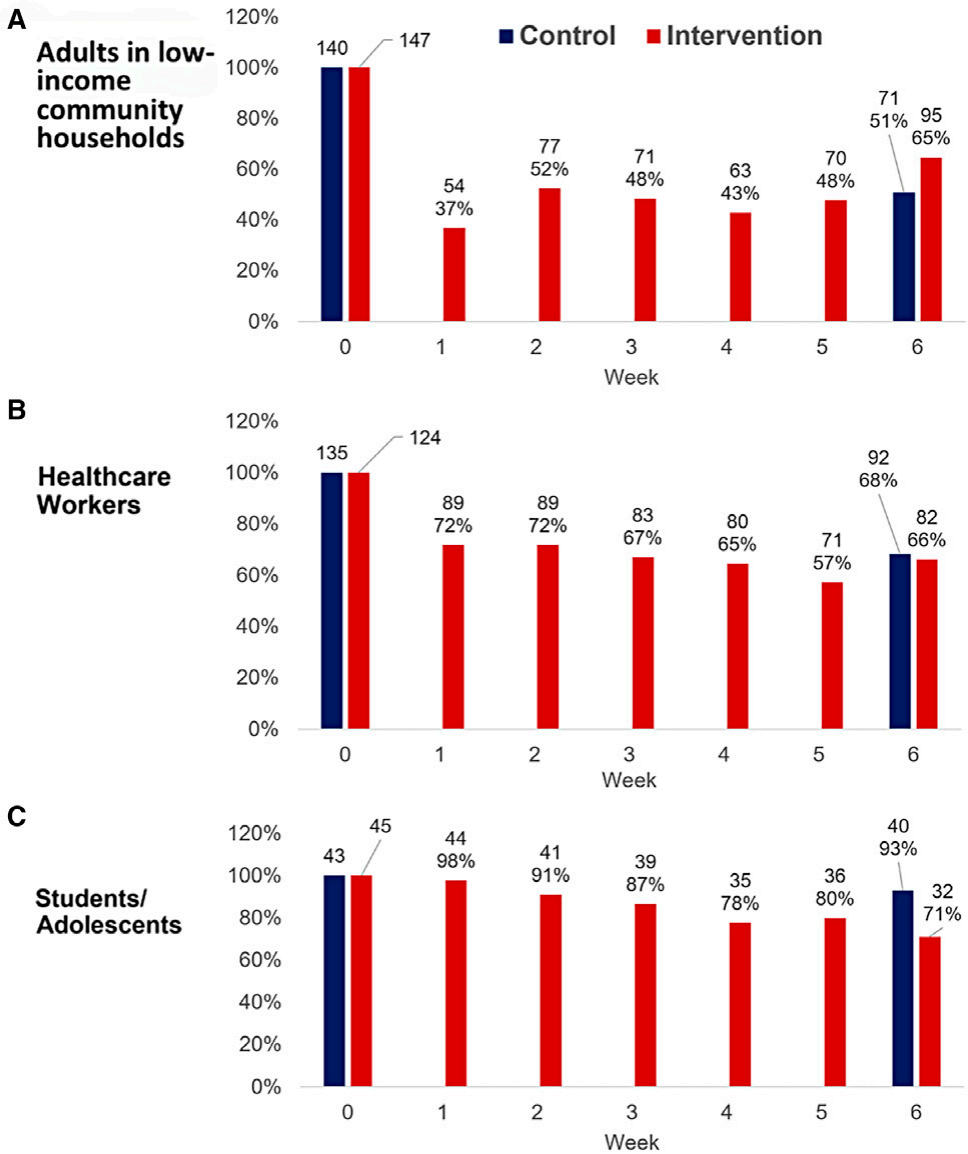
Ag-RDT testing across intervention and control communities.

Study follow-up was challenging across all three studies (Figure 2). In study A, 57% of participants; in study B, 66% participants; and in study C, 88% participants concluded follow-up. There were no statistically significant differences between intervention and control communities, and there was no difference between the different sexes attending the study visits for all three studies.

Follow-up was affected by 1) participants’ commitment to completing all follow-up visits, 2) work shift patterns (Study B), and 3) logistical factors. In study A, participants reported discomfort of testing and inconvenience as reasons for missed follow-up visits. Factors such as sex, use of public transport, and having an elderly person in the household did not consistently influence follow-up behavior across visits in this study.

Follow-up within these studies was also affected by 1) severe weather, which impacted accessibility and stability of temporary testing sites; 2) short-term subcontracting of staff, whose lack of commitment resulted in delayed testing at some testing sites; and 3) outbreaks of gang-related violence occurring in August Town during week 3 (Study A) and at one of the schools in week 6 (Study C). Study procedures were ceased during both outbreaks of violence to ensure continued safety of study participants and staff.

### Confirmed antigen and antibody positivity.

At baseline and at week six, all study participants were tested for SARS-CoV-2 using Ag-RDTs, PCR, and antibody RDT (ab-RDT). Overall, six participants (<1%) tested Ag-RDT positive during all three studies, including one person at baseline, one person at week 5, and two additional individuals at week 6 in study A. In study B, two people tested positive at baseline, and one of these remained positive for each of the subsequent visits. In study C, no one tested positive throughout the study. We did not detect any additional cases via PCR. The number of infections detected were hence too low to draw any conclusions relating to the efficacy of the intervention with regard to curbing SARS-CoV-2 transmissions within the studied communities where the testing took place. Overall, this aligns with the reduced number of new infections in the country at the time the studies were conducted.^7^

**Figure 3.**
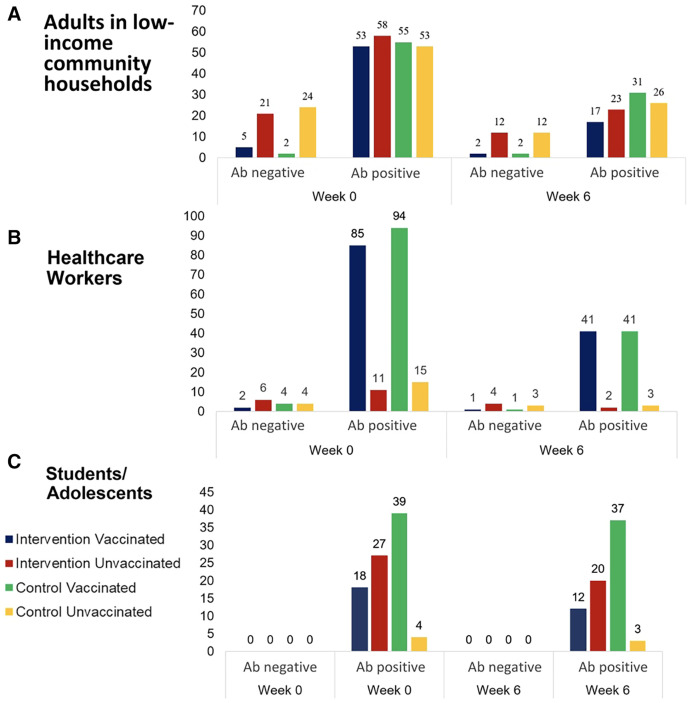
Number of individuals with detectable antibodies against SARS-CoV-2 at baseline and at week 6.

Anti-SARS-CoV-2 IgG antibody testing was conducted after all three studies had been concluded. For study A, antibody results were obtained for 94% (271/287) of the study participants (Figure 3A). At baseline, antibodies could be detected in 81% (219/271) of participants. Fifty-one percent (111/219) of those testing positive for SARS-CoV-2 antibodies had never been vaccinated, indicating past exposure to SARS-CoV-2. At week 6, antibody results were obtained for only 44% (125/287), and for the majority of these (78%, 97/125), antibodies against SARS-CoV-2 could be detected. Again, 51% (49/97) of those testing positive for SARS-CoV-2 antibodies had never been vaccinated.

For study B, antibody results were obtained for 84% (221/261) of the study participants (Figure 3B). At baseline, antibodies could be detected in 93% (205/221) of participants. At week 6, antibody results were obtained for only 37% (96/262), and for the majority of these (91%, 87/96), antibodies against SARS-CoV-2 could be detected. The majority of these participants were vaccinated.

For study C, antibody results were obtained for 88% (77/88) and 94% (83/88) of the study participants at baseline and week 6, respectively (Figure 3C). All participants tested, irrespective of their vaccination status, had detectable anti-SARS-CoV-2 IgG antibodies.

### Results of student survey.

**Figure 4.**
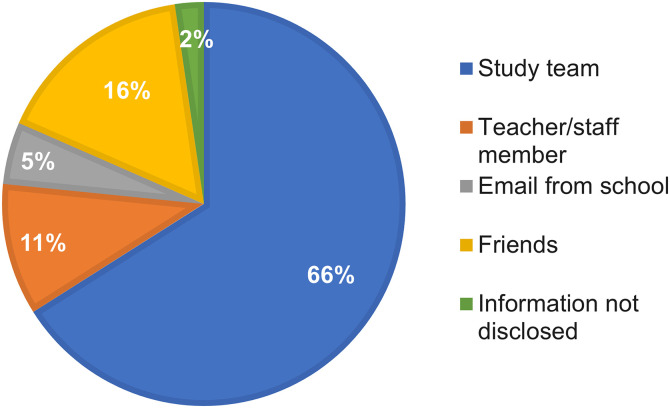
Source of information about study C (school study) for adolescents.

For study C, we explored various factors that may have influenced participation in this type of study as part of a structured survey. In total, 585 adolescents responded, including 43% (*n* = 253) female and 52% (*n* = 306) male adolescents. Twenty-six percent (4%; *n* = 26) did not disclose their sex. Most respondents (89%; *n* = 521) were aware that the study took place, and the majority of these had been alerted to the study by the study team (66%; Figure 4).

**Figure 5.**
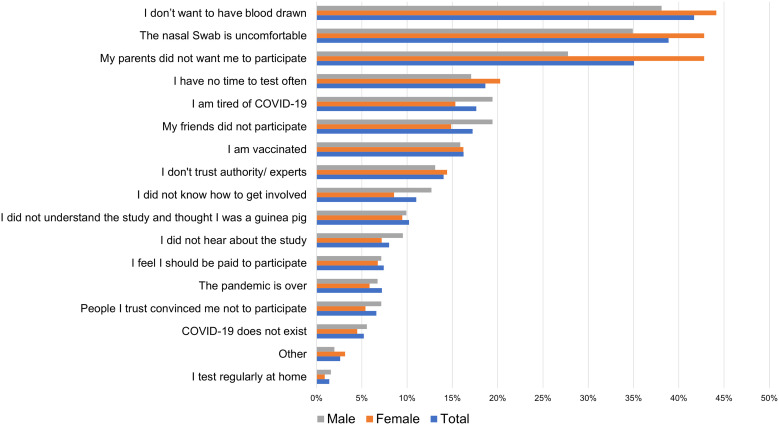
Reasons for not participating in study C.

Only 17% (86/521) of those aware of the study agreed to participate in the study, including 14% (*n* = 31) female and 20% (*n* = 52) male adolescents (*P* >0.07). Among those who had not been aware, 11% (*n* = 5) indicated that they would have participated, 32% (*n* = 14) were unsure, and 39% (*n* = 17) would not have participated. The remaining 18% (*n* = 8) did not disclose this information. The most common reasons for nonparticipation were 1) I don’t want to have blood drawn (42%; *n* = 208), 2) the nasal swab is uncomfortable (39%; *n* = 194), and 3) my parents did not want me to participate (35%; *n* = 175) (Figure 5).

We also explored the perception of the information provided by staff and the perception of the conduct of study staff. The majority (81%; 70/86) of study participants felt the study was well explained to them. The remainder did not feel well informed (9%; 8/86) or did not disclose the information (9%; 8/86). Of those who participated, 73% (*n* = 63) felt that the study team was friendly, and 60% (*n* = 52) felt that the team made them feel comfortable. Two male adolescents and no female adolescents felt that the team made them feel uncomfortable (2.3%).

**Figure 6.**
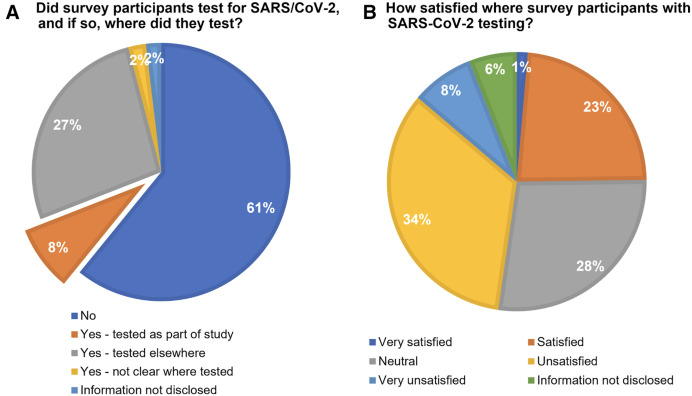
SARS-CoV-2 Ag-RDT testing.

Overall, 218 (37%) survey participants tested for SARS-CoV-2, either as part of the study (8%; *n* = 48), or elsewhere (Figure 6A). The testing experience was mixed (Figure 6B). There was no significant difference in sex with regard to whether participants tested, where participants tested, or their testing experience, and testing experiences were similar between study participants and those who tested elsewhere.

Only 53% of the survey respondents reported to have been vaccinated, but this was higher among those survey respondents who tested (71%, *P* <0.001). The following were the main reasons why survey participants did not get vaccinated: 1) I don’t trust it (57%, *n* = 158); 2) I think it’s harmful (12%, *n* = 34); 3) I don’t see others around me taking it (10%, *n* = 27); and 4) I was not informed about it (4%, *n* = 11). We focused on keeping the survey brief and hence did not explore these responses in more detail. There was no link between sex and vaccination rates or sex and the reasons for vaccine hesitancy.

## DISCUSSION

We applied the same testing strategy across three different high-risk populations, namely adults in low-income community households (Study A), healthcare workers (Study B), and adolescents attending secondary schools (Study C). Studies A and C occurred in low-income and volatile communities and study B in a major hospital in Jamaica. To the best of the authors’ knowledge, no similar study has been conducted in Jamaica, but various community-based COVID-19 testing strategies have been applied in other settings. Nonetheless, conducting repeat testing against SARS-CoV-2 with COVID-19 Ag-RDT proved challenging in all high-risk populations in which the testing strategy was assessed. Although the studies were not designed to compare the impact of the testing approach between the different communities, we learned several key lessons during their conduct.

### Key lessons.

1. Mobilizing participants was difficult for all three studies and proved most challenging for adolescents. As per a structured survey conducted as part of study C, less than one-fifth of the adolescents who were invited to participate joined the study, and one-third of participants declined participation because of lack of a parent’s consent. This highlights that significant barriers would need to be addressed if regular testing of adolescents was to be implemented as a public health measure.
2. All students participating study C had anti-SARS-CoV-2 IgG antibodies, irrespective of vaccination status, indicating that in Jamaica this population had high exposure to SARS-CoV-2 during the pandemic. Vaccination rates differed significantly between both control and intervention communities (91% versus 40%). Understanding the difference in vaccination rates between these two student communities is important because, given the low participation and follow-up rate, mass vaccination may present a more suitable public health intervention than regular testing.
3. Safety and security will need to be considered when choosing locations for community interventions. Collaborations with civil security/police forces and regular engagement with senior leaders of the community would likely be beneficial in minimizing the effect of violence on future studies. Community leaders may be able to leverage their influence on the younger, more criminally indisposed members to allow for a “holiday period” of limited criminal activity as public health interventions are assessed and/or implemented.
4. Jamaica is prone to very windy conditions, and solid structures, such as community venues or mobile vans would likely fare better than tents when used for community-based testing strategies.
5. Community-based interventions require a committed, well-versed, and competent workforce to ensure consistency. Establishing this workforce takes time and is likely even more difficult to mobilize and retain in outbreak situations, potentially leading to redistribution of staffing resources from healthcare facilities. Subcontracting some services, such as driving to other companies, proved challenging largely because of the lack of commitment and short notice or same-day cancellations.
6. Research in Jamaica, particularly in the medical field, generally uses less invasive data collection methods, such as reviews, questionnaires, and focus groups. As such, our implementation of this relatively novel research approach was met with experiences of hesitation and distrust among participants. Therefore, a normalization of this type of research study may be beneficial through extensive community engagement activities, public awareness and more similar research being done to build trust, dispel myths, and encourage participation.

### Recommendations.

We developed several recommendations as part of these studies to better prepare for future outbreak situations.

1. Healthcare providers should provide consistent messaging with regard to prevention and control measures such as testing, vaccination, and nonpharmaceutical interventions, including community engagement officers, local community leaders, traditional media (e.g., radios), digital media, and billboards.
2. Free testing should be swiftly implemented and coupled with prevention tools and strategies such as free masks and hand sanitizers in low-income communities and other public spaces that people frequent, such as bus parks, markets, and town centers.
3. Community leaders and security forces should be engaged to ensure that testing sites are safe and accessible for staff and to encourage those seeking healthcare.
4. Policies should be developed that take cultural sensitivities into account and can be easily adapted to outbreaks with similar transmission dynamics.
5. When conducting intervention studies, the staff should be appropriately trained to foster trust and comfort among potential participants and use creative strategies aimed at improving potential participants’ understanding to encourage participation.
6. The ethical review process needs to be improved to allow for swift evaluations of operational research studies, thereby allowing us to provide timely and relevant evidence to policy makers in Jamaica.

### Study limitations.

Several factors limited the of the testing procedures proposed as well as the outcomes of the three different studies.

1. Recruitment into the studies was challenging because of the general lack of trust in healthcare providers and government-related initiatives or activities, pandemic fatigue across the different communities, and the novelty of this type of research. This was compounded by the low SARS-CoV-2 transmission rates during the studies, which not only impacted our ability to evaluate the impact of our testing strategies fully, but also reduced the urgency of testing for individuals.
2. Follow-up within the study was challenging, partly because of pandemic fatigue and partly because the work was conducted within neglected communities (Studies A and C) that often had low health literacy. For study B (healthcare workers), changes to shift schedules and working hours, as well as reassignments to hospitals, also affected follow-up rates.
3. Studies A and C could not be completed because of outbreaks of severe violence, which resulted in some operations having to be ceased in both studies to ensure the welfare of study participants and study teams. During the outbreak of violence within the low-income communities, a state of emergency was declared, and the resulting restriction of movement and curfews also reduced transmission rates because of reduced social interactions within these communities. A shooting near one of the schools in study C during week 5 (October 25, 2022) resulted in limited operations, affecting both the control and intervention group.
4. We did provide transport reimbursement in study A to ensure that follow-up visits were not associated with any costs to participants. Participants in studies B and C received some refreshment for the inconvenience of repeated testing.
5. Due to the inherent biases of enrolling participants in each group and the timing, the seropositivity proportions are not directly comparable among groups.

## CONCLUSION

Our research highlighted significant social and economic challenges in conducting research in neglected communities in Jamaica. Factors such as crime, violence, stakeholder motivation, trust in healthcare, and environmental issues significantly influenced participant follow-up and impacted our findings. We urgently need to address these barriers to research and health within these populations to achieve greater equity in access to healthcare and in health outcomes.
